# Resting-State Connectivity of the Left Frontal Cortex to the Default Mode and Dorsal Attention Network Supports Reserve in Mild Cognitive Impairment

**DOI:** 10.3389/fnagi.2017.00264

**Published:** 2017-08-07

**Authors:** Nicolai Franzmeier, Jens Göttler, Timo Grimmer, Alexander Drzezga, Miguel A. Áraque-Caballero, Lee Simon-Vermot, Alexander N. W. Taylor, Katharina Bürger, Cihan Catak, Daniel Janowitz, Claudia Müller, Marco Duering, Christian Sorg, Michael Ewers

**Affiliations:** ^1^Institute for Stroke and Dementia Research, Klinikum der Universität München, Ludwig-Maximilians-Universität München Munich, Germany; ^2^Department of Diagnostic and Interventional Neuroradiology, Klinikum Rechts der Isar, Technische Universität München Munich, Germany; ^3^TUM-Neuroimaging Center of the Klinikum Rechts der Isar, Technische Universität München Munich, Germany; ^4^Department of Psychiatry and Psychotherapy, Klinikum Rechts der Isar, Technische Universität München Munich, Germany; ^5^Department of Nuclear Medicine, University of Cologne Cologne, Germany; ^6^German Center for Neurodegenerative Diseases (DZNE, Bonn) Bonn, Germany; ^7^Department of Psychology, University of Southampton Southampton, United Kingdom; ^8^German Center for Neurodegenerative Diseases (DZNE, Munich) Munich, Germany

**Keywords:** cognitive reserve, mild cognitive impairment, frontoparietal control network, memory, functional connectivity

## Abstract

Reserve refers to the phenomenon of relatively preserved cognition in disproportion to the extent of neuropathology, e.g., in Alzheimer’s disease. A putative functional neural substrate underlying reserve is global functional connectivity of the left lateral frontal cortex (LFC, Brodmann Area 6/44). Resting-state fMRI-assessed global LFC-connectivity is associated with protective factors (education) and better maintenance of memory in mild cognitive impairment (MCI). Since the LFC is a hub of the fronto-parietal control network that regulates the activity of other networks, the question arises whether LFC-connectivity to specific networks rather than the whole-brain may underlie reserve. We assessed resting-state fMRI in 24 MCI and 16 healthy controls (HC) and in an independent validation sample (23 MCI/32 HC). Seed-based LFC-connectivity to seven major resting-state networks (i.e., fronto-parietal, limbic, dorsal-attention, somatomotor, default-mode, ventral-attention, visual) was computed, reserve was quantified as residualized memory performance after accounting for age and hippocampal atrophy. In both samples of MCI, LFC-activity was anti-correlated with the default-mode network (DMN), but positively correlated with the dorsal-attention network (DAN). Greater education predicted stronger LFC-DMN-connectivity (anti-correlation) and LFC-DAN-connectivity. Stronger LFC-DMN and LFC-DAN-connectivity each predicted higher reserve, consistently in both MCI samples. No associations were detected for LFC-connectivity to other networks. These novel results extend our previous findings on global functional connectivity of the LFC, showing that LFC-connectivity specifically to the DAN and DMN, two core memory networks, enhances reserve in the memory domain in MCI.

## Introduction

The reserve theory proposes that individuals with favorable cognitive and lifestyle factors such as education, IQ and occupational complexity can transiently maintain relatively high cognitive performance when developing Alzheimer’s disease (AD), the most common cause of dementia in elderly people (Stern, 2012). While results from epidemiological studies have revealed protective factors in aging (Valenzuela and Sachdev, 2006), the neural underpinnings of reserve are still poorly understood. Recently, it was suggested that the fronto-parietal control network plays a crucial role in maintaining mental health and cognition in psychiatric and neurodegenerative diseases (Cole et al., 2014b). Supporting this, we recently found in patients with mild cognitive impairment (MCI) that higher resting-state global functional connectivity of the fronto-parietal control network (Franzmeier et al., 2016a), in particular a hub in the left lateral frontal cortex (LFC, Brodmann Area 6/44, also referred to as inferior frontal junction), is associated with more years of education. Furthermore, higher global LFC-connectivity was associated with a less severe reduction in memory performance at a given level of AD-induced FDG-PET hypometabolism (Franzmeier et al., 2017b), suggesting that LFC-connectivity may underlie previously observed protective effects of education in elderly people with emerging AD pathology (Ewers et al., 2013; Soldan et al., 2013). The fronto-parietal control network is task-invariantly involved in cognition (Duncan, 2010), and shows high connectivity to other networks that are engaged during a particular task (Cole et al., 2013; Helfrich and Knight, 2016). The LFC hub shows strong positive connectivity to fronto-parietal and dorsal attention subnetworks of the control network among other networks, but negative connectivity (anti-correlated) to the default mode network (DMN) (Cole et al., 2012). Since these networks have previously been implicated in episodic memory (Chai et al., 2014; Franzmeier et al., 2017a), it is possible that the global LFC connectivity to those networks is of particular importance for supporting reserve of memory abilities in AD. Thus, rather than assessing global LFC-connectivity at the whole-brain level as done previously (Cole et al., 2012; Franzmeier et al., 2017b), we assessed here systematically the global connectivity of LFC to other resting-state networks as a predictor of higher memory reserve in MCI.

The major aim of the current resting-state fMRI study was to test our hypotheses that (1) higher education is associated with higher LFC-connectivity to other major resting-state functional networks, and (2) higher connectivity between the LFC and other networks is associated with higher memory-related reserve. To address the questions set out by the current study, we computed the global LFC-connectivity to seven major brain networks (i.e., fronto-parietal, limbic, dorsal-attention, somatomotor, default-mode, ventral-attention, visual) (Yeo et al., 2011). Estimation of centrality measures such as resting-state global connectivity shows a relatively high reliability especially among heteromodal brain regions such as the LFC and may thus constitute a feasible measure to study neural mechanisms of reserve (Liao et al., 2013; Zuo and Xing, 2014). Since addressing these hypotheses on LFC to network connectivity required multiple testing, we examined all hypotheses in two independent sets of HC and MCI patients to independently validate our findings. To estimate memory-related reserve, we used a recently developed approach that captures the core of the reserve concept, i.e., how well-preserved memory performance is when accounting for the underlying level of brain pathology (Reed et al., 2010; Zahodne et al., 2013). In brief, we computed the difference between memory performance predicted by the level of age and brain pathology (i.e., hippocampal atrophy) and the actual level of memory performance, which is indicative of an individual’s reserve in the memory domain. We then tested whether higher LFC-connectivity to other networks predicted greater memory-related reserve in MCI. The results of the current study may help further our understanding on whether interactions between the LFC and specific functional networks support reserve in AD.

## Materials and Methods

### Subjects

For the current study we included two independent samples, each comprising both amnestic MCI subjects and healthy controls (HC). The first sample was recruited at the Technische Universität München (TUM) in Munich Germany (henceforth referred to as TUM sample) and encompassed 24 MCI patients and 16 HC. Here, MCI was diagnosed following the recommendations of the National Institute on Aging and the Alzheimer’s Association (Albert et al., 2011). All MCI patients included in the TUM sample met research criteria for prodromal AD, i.e., elevated levels of Amyloid-beta deposition (Aβ) as assessed via PiB-PET (Albert et al., 2011). Detailed descriptions of diagnostic and PET procedures have been reported previously (Koch et al., 2015). HC subjects showed no elevated Aβ levels and normal cognitive performance (i.e., CDR = 0; all CERAD-Plus scores not more than 1 SD below age- and gender-adjusted norms).

The second sample comprised 23 MCI and 32 HC recruited at the Institute for Stroke and Dementia Research (ISD sample), Ludwig-Maximilian University of Munich. Here, MCI was diagnosed according to the Petersen criteria (Petersen, 2004; Petersen et al., 2014), i.e., scoring 1.5 standard deviations below the age and gender-adjusted norms on at least one of the memory subtests of the CERAD-Plus battery (Schmid et al., 2014). HC subjects showed no cognitive symptoms (all CERAD-Plus scores not more than 1.5 SD below age-, gender- and education adjusted norms). Details on inclusion criteria and diagnostic procedures in this sample have been reported previously (Franzmeier et al., 2016a). Patients of both samples underwent structural MRI, resting-state fMRI as well as neuropsychological testing using the CERAD-Plus battery (Schmid et al., 2014).

Both studies were approved by the ethics committees of the respective institutions and conducted in accordance with the 1964 Helsinki declaration and its later amendments. Written informed consent was obtained from all subjects.

### MRI Acquisition

#### TUM Sample

Scanning was performed on a Philips 3T MRI scanner system using an 8-channel phased-array head coil. A structural high-resolution T1-weighted MPRAGE image was recorded with an isotropic voxel resolution of 1mm. fMRI data were recorded using a gradient EPI sequence with a TR/TE = 2000/35 ms, a flip angle of 82°, with an in-plane resolution of 2.75 mm and a slice thickness of 4 mm without an interslice gap. The resting-state fMRI scan comprised a total of 300 volumes, during which subjects were instructed to keep their eyes closed and not to fall asleep.

#### ISD Sample

All scans were acquired on a Siemens Verio 3T MRI scanner using a 32-channel head coil. Initially, a structural image was obtained using a high-resolution T1-weighted MPRAGE sequence with 1mm isotropic voxels. Resting-state fMRI data were recorded using a EPI pulse sequence with a TR/TE = 2580/30 ms, a flip angle of 80° and 3.5 mm isotropic voxel resolution without an interslice gap. The overall scan comprised 180 volumes prior to which subjects were instructed to keep their eyes closed.

### Spatial Normalization of MRI Images

Preprocessing of MRI data was conducted separately for both samples but using the same SPM12-based protocol (Wellcome Trust Centre for Neuroimaging, University College London, United Kingdom^1^). For each subject, high resolution T1-weighted structural images were segmented into probabilistic grey matter (GM), white matter (WM) and cerebrospinal fluid (CSF) maps via the new-segment approach implemented in SPM12 (Ashburner and Friston, 2005). For spatial normalization, we used DARTEL, a non-linear high-dimensional diffeomorphic registration algorithm that defines a group-specific template by warping each subject’s probabilistic tissue maps to a template space that is defined in an iterative procedure (Ashburner, 2007). This group-specific template was then affine-registered to an MNI template that is implemented in the DARTEL toolbox. Next, the non-linear and the affine transformation parameters were combined and applied to each subjects’ segmented tissue probability maps to achieve spatial normalization to MNI space. To define a group specific GM mask for each sample, the subject-specific spatially-normalized GM maps were averaged and binarized at a voxel value >0.3. In an equivalent step, we averaged and binarized the spatially-normalized WM (binarized at a voxel value >0.9) and CSF (binarized at a voxel value >0.7) maps that were required for denoising of the resting-state fMRI data.

### Hippocampal Volume Assessment

As a surrogate for neuronal loss that is highly related to AD pathology and to memory impairment in MCI (Petersen et al., 2000) we assessed the volume of the bilateral hippocampi using a previously described approach that yields highly similar results as manual hippocampal segmentation but has the advantage of being fully automated (Mak et al., 2011). Using the DARTEL flow-fields that were estimated during spatial normalization, we normalized the subject specific GM maps to MNI space and applied an 8 mm full width at half maximum (FWHM) Gaussian smoothing kernel. During the normalization step, modulation was applied to each image, to preserve local GM concentrations while warping the image to MNI space (Good et al., 2001). Each subjects’ normalized and modulated GM map was subsequently masked with a bilateral hippocampus mask selected from the widely used Automatic Anatomic Labeling atlas (Tzourio-Mazoyer et al., 2002). From these masked images we then extracted the bilateral hippocampal volume (Jack et al., 2000; Petersen et al., 2000).

### Preprocessing of Resting-State fMRI Data

Preprocessing was conducted using SPM12 again separately for both samples but following the same protocol. In a first step, we discarded the first 10 volumes from each resting-state session due to known stabilization effects of the magnetic field. All remaining EPI volumes were realigned to the first volume and subsequently coregistered to the high-resolution T1-weighted images in native space. None of the scanned subjects showed excessive head motion (translation: >3 mm; rotation: >3°). We further tested whether diagnostic groups (HC vs. MCI) showed differences in head motion. To this end we computed the average frame-wise displacement for each individual following a previously described protocol (Power et al., 2014). When comparing the average frame-wise displacement across diagnostic groups using a two-sample *t*-test, we found no group differences between HC and MCI [ISD: *t*(54) = -0.790, *p* = 0.433; TUM: *t*(39) = -1.664, *p* = 0.104]. For spatial normalization to MNI space, the non-linear DARTEL and affine registration parameters that were estimated during preprocessing of the T1-weighted images were combined and applied to the coregistered EPI volumes. All EPI images were subsequently smoothed using an 8 mm FWHM Gaussian kernel, detrended and band-pass filtered, using a frequency band of 0.01–0.08 Hz. We further regressed out the 6 motion parameters (3 translations, 3 rotations) and the BOLD signal averaged across the WM and CSF masks that were created during preprocessing of the T1-weighted images. We did not apply global signal regression since it can artificially introduce anti-correlations in the BOLD signal (Murphy et al., 2009; Murphy and Fox, 2016).

### Definition of the LFC Seed Region Associated with Reserve

**FIGURE 1:**
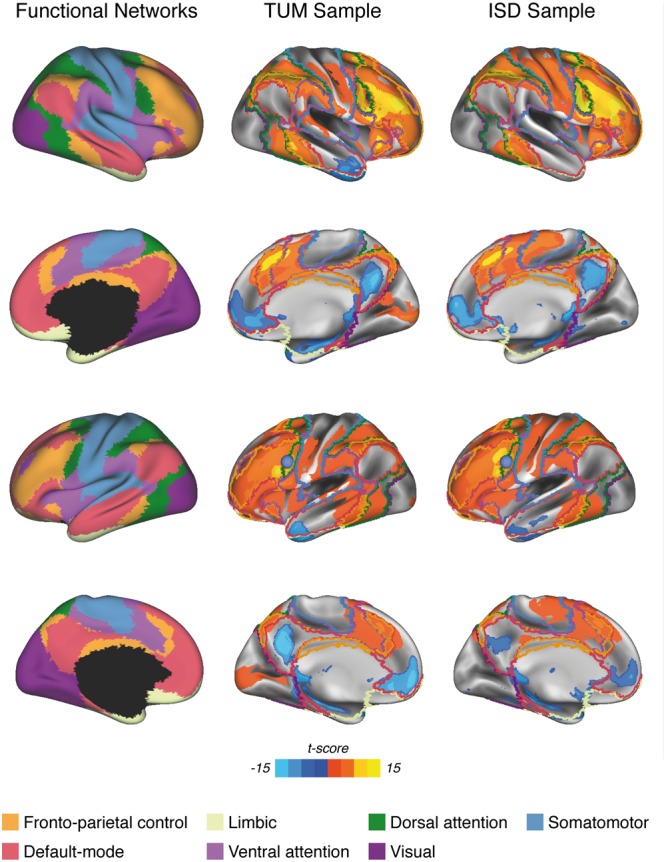
Surface renderings of significant LFC-connectivity in the TUM sample and the ISD sample (pooled across diagnostic groups) at a voxel threshold of α < 0.001, FWE-cluster-corrected at α < 0.05, superimposed on the functional network parcellation that was used for the current analyses. The LFC seed-ROI is superimposed as a blue sphere on surface renderings of the left hemisphere (3^rd^ row).

The location of the LFC ROI was based on our previous study in patients with MCI due to AD, where we showed that greater resting-state global LFC-connectivity was associated with more years of education and allowed to maintain memory performance relatively well in the face of AD-related posterior parietal FDG-PET hypometabolism (Franzmeier et al., 2017b). In brief, the previous study determined the LFC seed ROI as the meta-analytical peak coordinate of brain activation associated with cognitive control (Yarkoni et al., 2011). The LFC ROI was created as a spherical ROI with 8 mm radius centered around that peak coordinate (Brodmann area 6/44; MNI: *x* = -42, *y* = 6, *z* = 28; see **Figure 1** for the ROI location), and used as a seed region for all subsequent functional connectivity analyses.

### Resting-State Functional Connectivity Analysis

All functional connectivity analyses were conducted for each subject using the algorithms of the REST toolbox (Song et al., 2011). In a first step, we aimed to explore the spatial pattern of resting-state LFC-connectivity to confirm previous observations that the LFC is positively connected to fronto-parietal brain regions and negatively connected to midline regions belonging to the DMN (Spreng et al., 2010, 2013; Cole et al., 2012; Franzmeier et al., 2017b). To this end, we computed the Pearson-moment correlation between the LFC ROI and each voxel falling within the group specific GM masks. All correlations were subsequently Fisher-z transformed and saved as a 3D functional connectivity map. From these 3D functional connectivity maps, we excluded voxels belonging to the binary LFC ROI to avoid including any autocorrelations in later analyses. Next, we assessed the average connectivity of the LFC to 7 canonical networks that have been reported previously (Yeo et al., 2011) (i.e., DMN, DAN, ventral attention network, somatomotor, visual, fronto-parietal control, limbic). To avoid sample specific bias in network definitions, all network boundaries (see **Figure 1**, left panel) were defined independent of the current study based on a widely used brain network parcellation scheme assessed on 1000 subjects (Yeo et al., 2011). The downloaded 3D binary network maps were additionally masked with the group specific gray matter mask for each sample. To assess network specific LFC-connectivity for each subject, we then averaged the connectivity values (i.e., Fisher z-transformed correlations) across voxels that fell within each of the network masks. In an additional exploratory analysis, we repeated all above delineated steps, this time using a more fine-grained network parcellation that divides the 7 networks into 17 sub-networks (see **Supplementary Figure S1** for network definitions) (Yeo et al., 2011).

### Assessment of Memory Reserve

**FIGURE 2:**
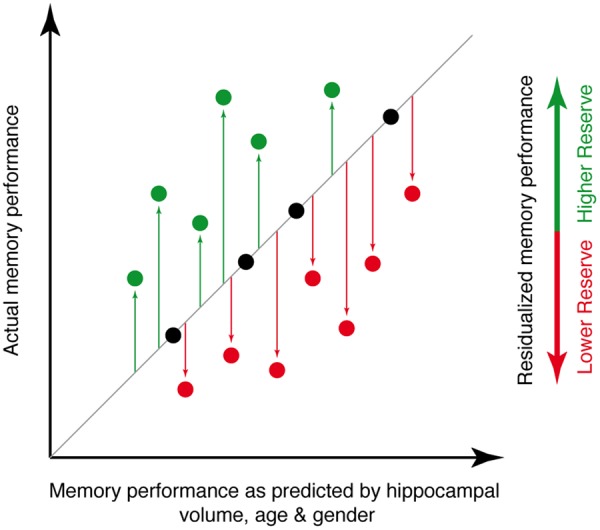
Illustration of the principle underlying the memory reserve measure used for the current study. The actual level of memory performance is plotted against the memory performance as predicted by age, gender and hippocampal volume. Individuals whose actual memory performance level is higher than predicted (green circles) have high memory reserve, whereas individuals whose actual memory performance is lower than predicted (red circles) have low memory reserve.

As a measure of memory performance, we used the delayed free recall subscale of the word-list learning test included in the CERAD-Plus battery. The test includes a list of 10 unrelated words for examining memory and is thus suitable for older individuals and cognitively impaired patients for whom longer lists would be too taxing. The words are shown at a rate of 2 s each and presented in a different order in three learning trials. The tested individual is instructed to read out every word to ensure word registration. After each learning trial, the tested individual is asked to recall the list. After a 3 to 5 min delay, retention is tested by free recall (Schmid et al., 2014). Using this free recall score, we assessed memory reserve via a decomposition of episodic memory variance following a modified approach that was introduced previously (Reed et al., 2010; Zahodne et al., 2013). In brief, we determined to what extent an individuals’ memory performance was better or worse than expected based on age, gender and hippocampal volume. To compute the memory reserve score, we applied linear regression in each sample using the word-list delayed free recall score as a dependent variable and regressed out variance explained by hippocampal volume, age and gender. Regression residuals were used as a measure of memory reserve. **Figure 2** illustrates the principle of this residualized reserve measure.

### Statistical Analysis

For each sample, group demographics, cognitive scores and hippocampal volume were compared between MCI and HC using two-sample *t*-tests for continuous measures and Chi-square tests for categorical measures. To map the spatial pattern of LFC-connectivity and to assess whether the LFC exhibits connectivity predominantly to the fronto-parietal brain regions and the DMN similar to previous reports (Spreng et al., 2010, 2013; Cole et al., 2012), we mapped significant LFC-connectivity for each sample (pooled across MCI and HC) in a voxel-wise manner using one-sample *t*-tests against zero with a voxel threshold of α = 0.001 and a FWE cluster threshold of α = 0.05, to correct for multiple comparisons on the cluster level. To assess the similarity of LFC-connectivity between both samples (ISD vs. TUM) we computed the spatial correlation (Wen et al., 2012) and R^2^ between significant LFC-connectivity maps, and additionally the dice similarity index (Zou et al., 2004) on binarized thresholded images (i.e., at a voxel threshold of α = 0.001 and a FWE cluster threshold of α = 0.05). Next, we tested our main hypotheses, that greater LFC-connectivity to fronto-parietal networks and the DMN are associated with greater memory reserve in MCI. This hypothesis was assessed on all seven canonical networks to test the specificity of effects for fronto-parietal networks and the DMN. To test our hypotheses, we used linear regression models to assess whether stronger LFC-connectivity to a network predicts higher memory reserve scores, i.e., residualized memory performance after accounting for age, gender and hippocampal volume. To avoid model estimation bias due to multicollinearity among predictors, we ran separate regression models for each of the 7 LFC to network connectivity measures as a predictor of memory reserve. Since our hypotheses clearly specified a directionality of effects (i.e., stronger LFC-connectivity is associated with higher memory reserve), we applied a one-tailed p-threshold in order to consider associations significant. To ensure that these results were not driven by spurious correlations, we repeated the above listed analyses when computing the average LFC to network connectivity only across those voxels that surpassed the group level *t*-test against 0 (as shown in **Figure 1**). We further tested whether associations between LFC-connectivity and memory reserve were specific to the stage of MCI or whether these associations also applied to HC. To this end, all models were computed in an equivalent fashion in the HC groups.

In an exploratory analysis, we repeated all above delineated analysis steps this time using the 17-network parcellation (**Supplementary Figure S1**) that divides the 7 networks into smaller sub-networks. The rationale for including this additional analysis was to test whether associations between memory reserve and LFC-connectivity to the DMN or DAN were driven by connectivity to specific sub-parcels of these networks.

In a last step, we aimed to extend previous findings [i.e., that higher global LFC-connectivity is associated with greater education as the most widely used reserve proxy (Stern, 2012; Franzmeier et al., 2017b)] to the current network-specific hypotheses. To this end we assessed whether more years of education were also associated with increased LFC-connectivity to those networks for which we found a significant association with memory reserve (DMN & DAN). Since education was only available in the ISD sample, this analysis was not independently validated. In brief, we computed linear regression models, with LFC-connectivity (DMN or DAN) as the dependent variable and education as the independent variable, controlling for age and gender.

All linear regression models were computed using the freely available statistical software package *R* (R Development Core Team, 2013). Linear model assumptions (skewness, kurtosis, heteroscedasticity) were tested using the gvlma function implemented in *R*. Normal distribution of residuals was assessed using a Shapiro–Wilk test. For all models reported, no significant (α = 0.05) violations of linear model assumptions were found.

## Results

**FIGURE 3:**
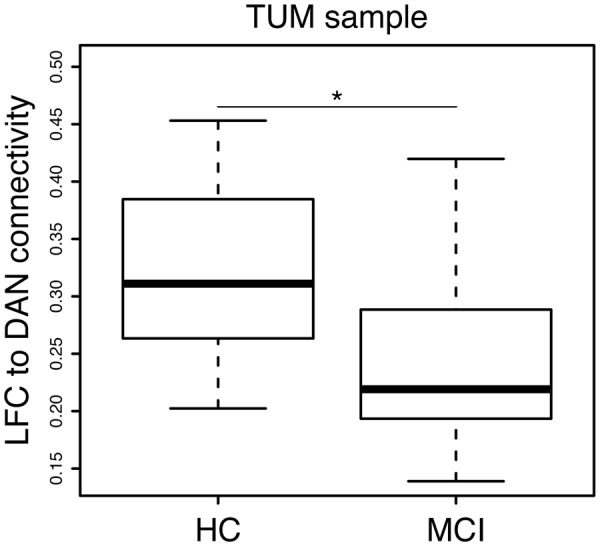
Significant group difference (MCI vs. HC) in LFC-connectivity to the DAN in the TUM sample.

Group demographics, cognitive scores and average LFC-connectivity measures are displayed in **Table 1**. In the ISD sample, the MCI group was significantly older than the HC group. Consistent across both samples, the MCI groups showed significantly lower MMSE and word-list delayed recall scores as well as lower hippocampal volume. In the TUM sample, LFC to DAN connectivity was significantly reduced in MCI when compared with HC (**Figure 3**).

**Table 1:** Sample characteristics.

	TUM		ISD	
	HC (*n* = 16)	MCI (*n* = 24)	HC (*n* = 32)	MCI (*n* = 23)
Age	65.25 (5.51)	68.50 (8.28)	71.27 (5.25)	75.68 (4.22)^1^
Gender (m/f)	7/9	14/10	14/18	9/14
Years of education	n.a.	n.a.	13.94 (3.06)	13.36 (3.59)
APOE genotype (𝜀4 carrier/non-carrier/not available)	7/6/3	14/8/2	9/23	11/11/1
MMSE score	29.12 (0.81)	26.96 (1.60)^2^	29.44 (0.80)	26.32 (2.38)^2^
CERAD word list delayed recall score	7.42 (1.68)^3^	4.08 (2.69)^2^	8.34 (1.43)	4.05 (2.24)^2^
LFC to DMN connectivity	–0.16 (0.05)	–0.15 (0.04)	–0.18 (0.07)	–0.16 (0.05)
LFC to DAN connectivity	0.32 (0.08)	0.24 (0.07)^2^	0.34 (0.09)	0.32 (0.09)
Hippocampal Volume (in ml)	5.85 (0.37)	5.23 (0.68)^2^	5.61 (0.63)	5.04 (0.76)^2^
				

*^1^MCI > HC, ^2^MCI < HC (*p* < 0.05); ^3^scores were only available for 12 HC subjects of the TUM sample.*

### Voxel-Wise Mapping of LFC-Connectivity

**Figure 1** shows the pattern of significant LFC functional connectivity, as assessed via voxel-wise one-sample *t*-tests pooled across diagnostic groups in each sample. Visual inspection of the figure reveals that the LFC exhibits significant positive connectivity to regions belonging to the DAN, fronto-parietal control network, VAN and somatomotor network as well as the visual network. Negative connectivity is found predominantly to midline regions belonging to the DMN, as well as inferior temporal brain regions (i.e., limbic network). Overall, this pattern of LFC connectivity is in line with previous research (Cole et al., 2012; Franzmeier et al., 2017b). Comparing the pattern of significant LFC-connectivity across both samples yielded a high similarity as indicated by a high spatial correlation coefficient (*r* = 0.81, *p* < 0.001, *R*^2^ = 0.66) and dice similarity index of 0.72. When recomputing the spatial correlation using a less restrictive threshold (voxel level α = 0.01, FWE cluster corrected at α = 0.05) thereby including a higher number of voxels showing lower connectivity, the correlation (*r* = 0.72, *p* < 0.001, *R*^2^= 0.52) and dice similarity index of 0.65 among both samples remained high.

### Higher LFC to DMN/DAN Connectivity Is Associated with Greater Memory Reserve in MCI

**FIGURE 4:**
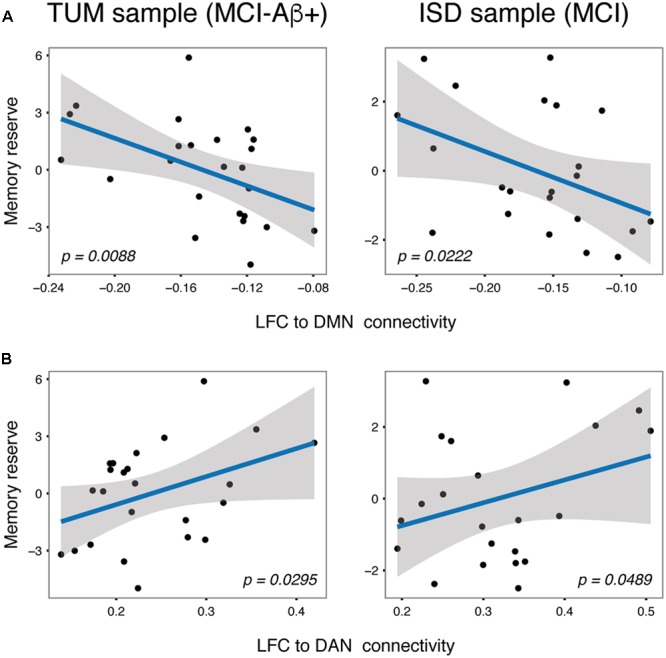
Scatterplots for the independently validated associations between LFC to DMN **(A)** and LFC to DAN **(B)** connectivity and memory reserve in MCI subjects.

Next we tested our hypothesis that higher LFC-connectivity to the DMN and DAN predicts greater memory reserve. For the DMN, results of the regression analysis showed that stronger (negative) connectivity between the LFC and the DMN was associated with higher memory reserve in MCI. This association was consistently detected for MCI patients of the TUM [*t*(22) = -2.569, B/SE = -31.23/12.16, *p* = 0.0088; **Figure 4A**, left panel] and ISD sample [*t*(21) = -2.138, B/SE = -16.18/7.57, *p* = 0.0222; **Figure 4A**, right panel]. When computing the average LFC-connectivity only to those voxels that surpassed the group level *t*-test against 0 (as shown in **Figure 1**), we obtained congruent associations between LFC-connectivity to the DMN and memory reserve [TUM sample: *t*(22) = -1.875, B/SE = -18.30/9.76, *p* = 0.0375; ISD sample: *t*(21) = -1.961, B/SE = -13.72/6.99, *p* = 0.0317]. In the HC groups, LFC to DMN connectivity was not associated with memory reserve, neither in the TUM sample [*t*(10) = -1.301, B/SE = -13.85/10.64, *p* = 0.111], nor when tested in the ISD sample [*t*(30) = -0.719, B/SE = -2.71/3.53, *p* = 0.239].

For the DAN, higher (positive) connectivity between the LFC and DAN was associated with greater memory reserve in MCI consistently across the TUM [*t*(22) = 1.992, B/SE = 14.69/7.38, *p* = 0.0295; **Figure 4B**, left panel] and ISD sample [*t*(21) = 1.737, B/SE = 7.44/4.28, *p* = 0.0489; **Figure 4B**, right panel]. When assessing LFC-connectivity to the DAN based on only those correlations that surpassed the group-level *t*-test against 0 (**Figure 1**), we obtained a similar result including significant effects of LFC to DAN connectivity on memory reserve [TUM sample: *t*(22) = 2.311, B/SE = 13.86/6.00, *p* = 0.0153; ISD sample: *t*(21) = 2.123, B/SE = 7.51/3.54, *p* = 0.0229]. Again, no associations between LFC to DAN connectivity and memory reserve were found for HC subjects [TUM: *t*(10) = 0.827, B/SE = 9.51/11.50, *p* = 0.2135; ISD: *t*(30) = -0.642, B/SE = -1.63/2.61, *p* = 0.269].

When testing whether LFC-connectivity to other networks (visual, somatomotor, fronto-parietal control, ventral attention, limbic; see **Figure 2** for network boundaries) predicted better memory reserve in MCI or HC, no significant associations were detected, neither for MCI nor HC of both samples (Supplementary Table S1). This result pattern remained unchanged when restricting the analysis to voxels that surpassed the group-level *t*-test against 0. Together, these results suggesting specificity of our findings for LFC-connectivity to the DMN and DAN as predictors of memory reserve. This was confirmed by our exploratory analysis, where we tested the association between memory reserve and LFC-connectivity to sub-parcels of the larger networks (**Supplementary Figure S1**). Here, we could show that greater (negative) LFC-connectivity to large medial and lateral parcels of the DMN (16 and 17 in **Supplementary Figure S1**) and greater (positive) LFC-connectivity to all parcels of the DAN (5 and 6 in **Supplementary Figure S1**) was predictive of memory reserve in MCI across both samples. Statistical details on these analyses can be found in Supplementary Table S2. Again, this result pattern remained unchanged when restricting the analysis to voxels that surpassed the group-level *t*-test against 0.

### LFC to DMN and DAN Connectivity Is Associated with Education in MCI

In a last step we aimed to extend previous findings, that LFC-connectivity to those networks for which we found significant associations between LFC-connectivity and memory reserve is associated with years of education as a common reserve proxy. As shown by our regression analyses, greater education predicted higher (negative) LFC to DMN [*t*(19) = -1.771, B/SE = -0.006/0.003, *p* = 0.046] and higher (positive) LFC to DAN connectivity [*t*(19) = 2.248, B/SE = 0.011/0.005, *p* = 0.018] in the ISD sample. In the HC group, no significant associations between education and LFC-connectivity were detected [LFC to DMN: *t*(28) = 0.795, B/SE = 0.005/0.005, *p* = 0.217; LFC to DAN: *t*(28) = -1.016, B/SE = -0.007/0.007, *p* = 0.159].

## Discussion

Our major findings were that in MCI greater LFC-connectivity specifically to the DMN as well as the DAN was associated with higher memory-related reserve, i.e., relatively high memory performance when accounting for the level of brain pathology. These findings suggest that resting-state connectivity levels of the LFC to particular other networks contributes to memory-related reserve in MCI.

We showed that the LFC exhibits widespread functional connectivity to fronto-parietal and DMN networks, where higher LFC activity was associated with higher activity within the DAN regions, and lower activity within the DMN. These results are consistent with previous findings of the LFC as a hub of the task-positive network including the DAN, which is anti-correlated with the DMN (Spreng et al., 2010, 2013; Cole et al., 2012).

Both the DMN as well as the DAN have been consistently shown to be fundamentally involved in memory (Buckner et al., 2008; Kim et al., 2010; Spreng and Grady, 2010; Kim, 2015), where higher within network connectivity and higher DMN-DAN anti-correlation was associated with higher memory ability in neurodegenerative diseases (Sorg et al., 2007; Mevel et al., 2011; Finke et al., 2013; Zhang et al., 2015; Meskaldji et al., 2016; Franzmeier et al., 2017a). A previous study assessing task-related functional connectivity of fronto-parietal hubs including the LFC, has reported that the fronto-parietal control network couples with networks such as the DAN and DMN during task-demands, which was suggested to facilitate adaptive task performance (Cole et al., 2013). Since previous evidence shows that task-related brain activation and task-related connectivity are correlated with resting-state connectivity (Cole et al., 2014a; Tavor et al., 2016), it is possible that higher resting-state LFC connectivity to the DMN and DAN is predictive of higher LFC coupling to these networks during memory tasks. Studies on effective connectivity showed that the LFC is the driving force controlling the activity of other brain networks such as the DAN and the DMN (Gao and Lin, 2012; Wen et al., 2013). Future studies assessing effective connectivity may thus address whether the effective connectivity of the LFC to other networks is linked to greater memory performance during task in AD.

For HC, we did not detect any associations between resting-state LFC-connectivity and memory-related reserve. Possible explanations include ceiling effects in memory performance levels in the HC subjects or absence of AD pathology and ensuing aggravated hippocampal atrophy. Note that the delayed recall memory measure that was used to compute the residualized memory reserve score is tailored for the clinical detection of cognitive impairment and may thus be less amenable to detect slight cognitive decline in cognitively asymptomatic controls. Thus the absence of the detection between an association of LFC-connectivity and residualized memory performance in the HC groups of the current study may have been due to ceiling effects. As an alternative explanation for the current results, in the HC groups neurodegeneration may not be advanced to a level at which memory maintenance becomes reliant on high levels of resting-state connectivity of the LFC hub. We point out, however, that when the same HC subjects in the ISD sample were challenged with a difficult face-name association task, task-related LFC-connectivity was associated with higher education and memory reserve as assessed on task performance levels (Franzmeier et al., 2017c). Thus, we conclude that high levels of LFC-connectivity become crucial for reserve once the brain is challenged.

A strength of the current study is that we found an identical pattern of associations between LFC-connectivity to the DMN and DAN and reserve in the memory domain in two independent samples and when using different network parcellations, which guards against overfitting of statistical models in a given sample and Type I error in the face of multiple testing. However, when interpreting the results of the current study, several caveats should be considered. Since education was only available in one of our samples (ISD sample), the associations between education and LFC-connectivity in MCI could not be independently validated. However, our current results are in line with the findings of our previous study based on data from the Alzheimer’s disease neuroimaging initiative (Franzmeier et al., 2017b), showing that greater education is associated with increased LFC-connectivity in MCI-Aβ+ subjects, where greater LFC-connectivity attenuated detrimental effects of parietal FDG-PET hypometabolism on memory. Thus, the association between LFC-connectivity and education in MCI in the ISD sample is unlikely to be spurious. Furthermore, we assessed LFC-connectivity and its association with reserve at the symptomatic stage in subjects at increased risk of AD. It remains to be demonstrated whether LFC-connectivity supports reserve already at the preclinical stages of AD including cognitively normal subjects with emerging Aβ pathology or subjective cognitive decline as well as at the more progressed AD stage when mild dementia is visible. Future studies should assess the role of LFC-connectivity also in those other stages of AD ranging from preclinical to dementia, where reserve effects were reported (Meng and D’Arcy, 2012; Ewers et al., 2013). Life-span studies could be informative about the trajectory of LFC connectivity changes both in normal and pathological aging. Previous lifespan studies in HC subjects have shown age-relate decline in the fronto-parietal control network (Geerligs et al., 2015) and brain hubs such as the LFC (Betzel et al., 2014; Zuo et al., 2017). Life-span studies could also address to what extent life experiences such as education or occupational attainment, i.e., factors associated with higher reserve, are predictive of inter-individual differences in the trajectories of LFC-connectivity. In the current study, no strong indication of pathological decreases in LFC-connectivity to the DMN and DAN were found, suggesting that LFC-connectivity is relatively spared in AD. Thus, the understanding of the pre-morbid trajectories of LFC-connectivity may be pivotal in order to predict reserve capacity at an individual level in neurodegenerative diseases including AD.

In order to establish LFC-connectivity as a functional measure of reserve, test–retest reliability of LFC-connectivity estimation is fundamental. Previous studies have reported that test–retest reliability of centrality measures such as global connectivity or regional homogeneity is highest in heteromodal regions like the LFC and in higher order networks such as the DAN and DMN (Liao et al., 2013; Zuo and Xing, 2014). However, we encourage future studies to specifically assess test–retest reliability of LFC-connectivity estimation across healthy and clinical populations to support its use as a functional measure of reserve.

Overall, our results provide novel insight in how the LFC supports reserve in MCI via connectivity to memory-related functional networks. These findings could act as a starting point to assess modifiability of LFC-connectivity via cognitive and pharmacological interventions or brain stimulation (Dhanjal and Wise, 2014; Drumond Marra et al., 2015; Franzmeier et al., 2016b). Previous studies support the concept that transcranial magnetic stimulation on the LFC can improve memory in MCI (Drumond Marra et al., 2015) and that pharmacological treatment with acetylcholinesterase inhibitors increases frontal resting-state connectivity (Zaidel et al., 2012) and task-related frontal brain activation (Dhanjal and Wise, 2014). Together, this renders the LFC a promising target for fostering reserve mechanisms, which holds potential for secondary prevention of AD.

## Author Contributions

NF, study concept and design, data collection, statistical analysis, interpretation of the data, and drafting the manuscript. JG, TG, AD, MÁ-C, AT, LS-V, KB, CC, DJ, CM, MD, CS: data collection and critically revising the manuscript. ME, study concept and design, interpretation of the data, and drafting the manuscript.

## Conflict of Interest Statement

Outside the submitted work TG reported having received consulting fees from Actelion, Eli Lilly, MSD; Novartis, Quintiles, Roche Pharma, lecture fees from Biogen, Lilly, Parexel, Roche Pharma, and grants to his institution from Actelion and PreDemTech. The other authors declare that the research was conducted in the absence of any commercial or financial relationships that could be construed as a potential conflict of interest.

## Footnotes

1 www.fil.ion.ucl.ac.uk/spm

## Supplementary Material

FIGURE S1 Surface rendering of the 17-network parcellation that divides the 7 networks into smaller sub-networks. Network affiliations corresponding to the 7-network parcellation are displayed for each parcel.
