# Supplementary figures and images for: Resting-State Connectivity of the Left Frontal Cortex to the Default Mode and Dorsal Attention Network Supports Reserve in Mild Cognitive Impairment
Source: Front Aging Neurosci. 2017 Aug 7;9:264. doi: 10.3389/fnagi.2017.00264 (PMC5545597; doi:10.3389/fnagi.2017.00264)

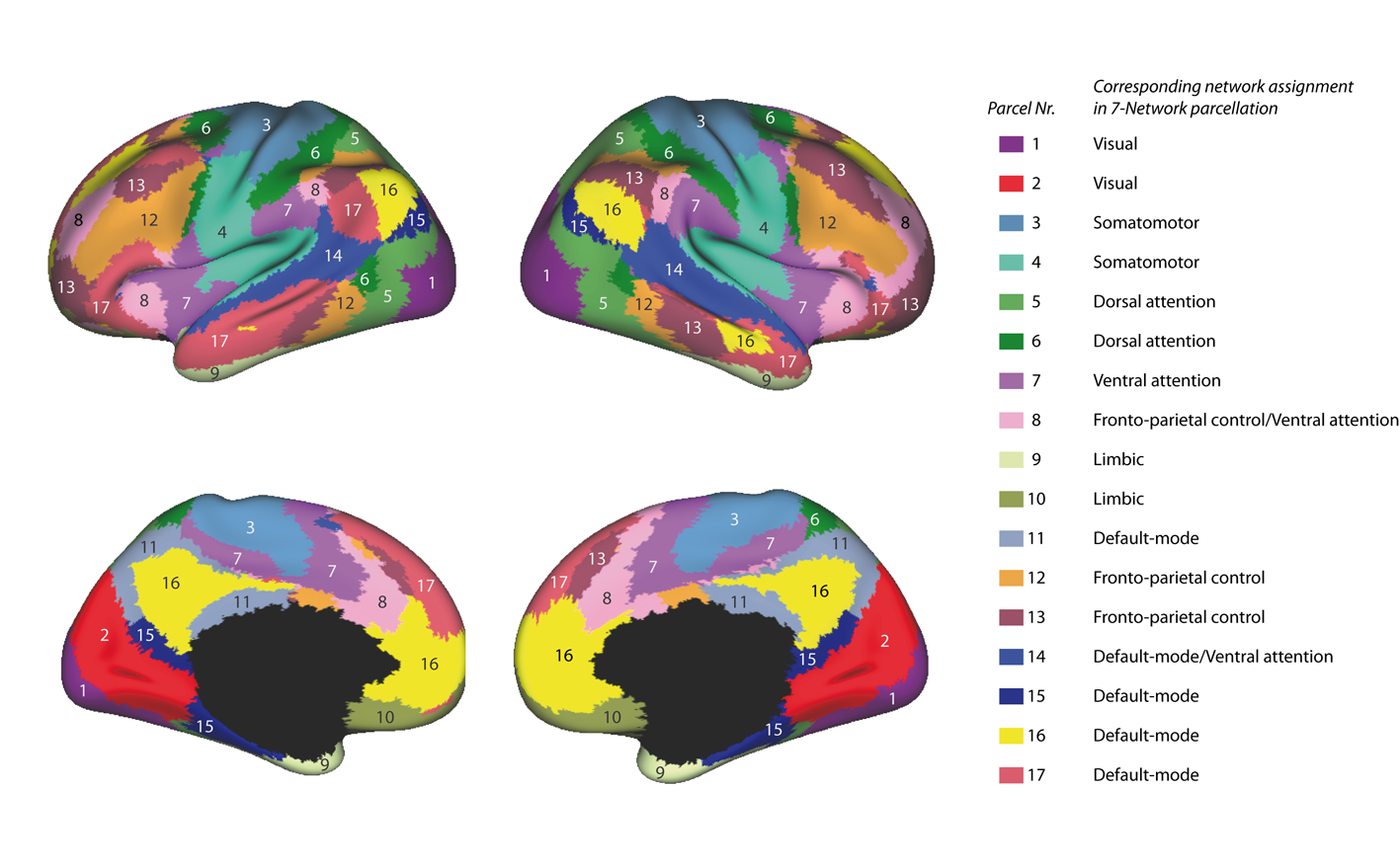

Supplement: FIGURE S1 — Surface rendering of the 17-network parcellation that divides the 7 networks into smaller sub-networks. Network affiliations corresponding to the 7-network parcellation are displayed for each parcel. [file Image_1.TIF]
